# Evaluation of cholestasis in Iranian infants less than three months of age

**Published:** 2015

**Authors:** Seyed Mohsen Dehghani, Neda Efazati, Iraj Shahramian, Mahmood Haghighat, Mohammad Hadi Imanieh

**Affiliations:** 1 *Gastroenterohepatology Research Center, Shiraz University of Medical Sciences, Shiraz, Iran*; 2 *Neonatology Research Center, Shiraz University of Medical Sciences, Shiraz, Iran *

**Keywords:** Cholestasis, Biliary atresia, Neonatal jaundice

## Abstract

**Aim**: The aim of this study is to find-out the possible etiologies in Iranian infants less than three months in Shiraz, South of Iran.

**Background**: Cholestatic jaundice most probably occurs due to a pathological condition and the most frequent causes in early infancy are neonatal hepatitis and biliary atresia. Early diagnosis and treatment of infantile cholestasis can improve prognosis of liver diseases by prevention of the complications of these disorders.

**Patients and methods**: In this retrospective study, 122 infants under 3 months of age with cholestasis were studied in Nemazee Hospital (affiliated to Shiraz University of Medical Sciences) during the years 2001-2011. Demographic data, duration of jaundice, liver biopsy and the causes of cholestasis were recorded.

**Results**: There were 76 males (62.3%) and 46 females (37.7%) with a mean age of 54.4 ± 23.7 days. The most common clinical finding was jaundice that was seen in all patients (100%).The onset of jaundice was the first day to the fifty two days of age, with an average age of 15.6 ± 16.1 days. Other findings included hepatomegaly in 92 patients (76.4%), clay-color stool in 54 (44.3%), and splenomegaly in 29 patients (23.8%). In this study, the most common causes of cholestasis were biliary atresia (30=24.6%), idiopathic neonatal hepatitis (30= 24.6%) and bile ducts paucity (16=10.3).

**Conclusion**: The results of this study showed that biliary atresia and neonatal hepatitis are the most common causes of infantile cholestasis in this area. It is recommended that biliary atresia should be discriminated from other forms of neonatal cholestasis.

## Introduction

Cholestasis results from structural and functional impairment of the hepatobiliary system and may manifest as a decline in physiologic bile flow, presence of bile pigment in hepatocytes and bile ducts, and clinically with collection of blood and extra hepatic tissues of substances excreted in the bile (1, 2).

The incidence of neonatal cholestasis is estimated around 1 in 2500 live births (3, 4). In infants, cholestasis associated with severe hepatic synthetic dysfunction points to life-threatening metabolic disorders. In this setting, early diagnosis and prompt treatment offer the only chance for survival. Fortunately, cholestasis in infants presents more frequently with initially normal liver synthetic function. Stepwise approach with initial step of rapid diagnosis of treatable disorders, such as sepsis, hypothyroidism, panhypopituitarism and inborn errors of metabolism must be determined and promptly treated. In the second step infants without evidence of counted causes, evaluation for patency of the extra-hepatic biliary system is a high priority because early surgical intervention results in a better outcome (1). 

The two most common causes are biliary atresia and idiopathic neonatal hepatitis (5, 6). Neonatal hepatitis is defined as intrahepatic cholestatic disorders other than structural disorders of the biliary tree (3). Idiopathic neonatal hepatitis is defined as intrahepatic cholestasis in which the characteristic giant-cell hepatitis lesion is present on liver biopsy but for which no cause (infectious, genetic, metabolic or anatomic) is identified (7). In light of the rapid advancement of BA to cirrhosis together with the curative effectiveness of early surgical intervention, attempting to prompt diagnostic testing in infants with conjugated hyperbilirubinemia should be considered (8). In hepatology center of Nemazee Hospital metabolic disorders (Wilson disease, hepatorenaltyrosinemia) cholestatic syndromes (biliary atresia, idiopathic neonatal hepatitis, progressive familial intrahepatic cholestasis) were the most common causes of cirrhosis in children (9). There is scarce data on neonatal cholestatic disorders in Iran, despiteearly detection of biliary atresia (BA) has a vital role in prevention of liver cirrhosis in these patients (10). Thus, the aim of this study is to find-out the possible etiologies in Iranian infants less than three months in Shiraz, South of Iran. 

## Patients and Methods

The medical records of 122 infants less than 3 months who were admitted in Pediatric Gastroenterology and Hepatology Department of Nemazee Hospital, Shiraz (referral center for pediatric liver transplant in Iran) with final diagnosis of cholestasis in 2001-2011 were reviewed. According to the guideline of the North American Society for Pediatric Gastroenterology, Hepatology, and Nutrition (NASPGHAN), a jaundice infant at 2 weeks of age was investigated for cholestasis by measuring serum bilirubin levels. A serum direct bilirubin level higher than 1mg/dL with a total bilirubin below than 5 mg/dL or beyond 20 percent of the total bilirubin if the total bilirubin was more than 5 mg/dL was considered indication for evaluation of cholestasis. Shiraz University of Medical Sciences Ethics Committee approved this research.

All infants with indirect hyperbilirubinemia due to causes such as Crigler–Najjar syndrome or hemolytic diseases and the infants more than 3 months of age at referral were excluded from the study. The study was conducted retrospectively by evaluating the patients’ medical records. All demographic data including age, sex, onset of jaundice, stool color, family history, abnormal liver function tests and liver biopsy (if done) for the diagnosis of cholestasis were recorded. Our approach followed three diagnostic steps. At first step, we established the presence of conjugated or direct hyperbilirubinemia (liver function test). In second step, we evaluated the patients for any evidences of systemic or metabolic diseases by history, physical examination, stool color, liver blood tests, complete blood counts, coagulation studies, urinalysis/urine culture, liver ultrasonography, and evaluation for galactosemia (urine reducing substances and GALT activity), cystic fibrosis (sweat chloride test), and hypothyroidism (T4 and TSH) was done for all patients and also TORCH works up if clinically indicated (low birth weight, intrauterine growth retardation, hepatosplenomegaly, and rash) and finally we differentiated biliary atresia from other intrahepatic causes of neonatal cholestasis (abdominal ultrasonography for triangular cored sign and absent or small< 1.5 cm or empty gallbladder, increased hepatic subcapsular flow on doppler ultrasonography, and hepatobiliary scintigraphy if the patient had clay color stool, liver biopsy and intraoperative cholangiography). Since, the golden time for Kasai operation in patients with biliary atresia is less than 2 months of age, if patient referred to our center at this age or older and clinically was suspected to biliary atresia or in which there was argue over diagnosis after review of imaging studies and liver biopsy for evaluation of patency of biliary tree we performed intraoperative cholangiography for saving the time. 

Chi-square test or Fisher's exact test was used for comparing categorical variables, and Mann-Whitney test was used for numerical variables between groups. Age differences between the groups were determined using ANCOVA test. 

## Results

Of 122 patients, 76 (62.3%) were male and 46 (37.7%) were female, and their mean age was 54.4±23.7 days. The biliary atresia (n=30; 24.6%), idiopathic neonatal hepatitis (n=30; 24.6%), and paucity of intrahepatic bile ducts (n=16; 13.1%) were the most common causes of cholestasis. The causes of cholestasis were unknown in 28 (22.9%) patients (table 1).

**Table 1:** Differences between the cause of cholestasis in males and females

Total (%)	*p*- value	Female	Male	Causes of Cholestasis
24.6	0.58	17	13	Biliary atresia
24.6	0.1	10	20	Idiopathic neonatal hepatitis
10.3	0.45	6	10	Intrahepatic bile duct paucity
8.2	0.38	4	6	Genetic and Metabolic causes
4.1	0.37	4	1	Progressive familial intrahepatic cholestasis
1.6	-	0	2	CMV cholestasis
0.8	-	0	1	Hepatic vein occlusion
22.8	0.14	5	23	Unknown causes

In this study, more cases of biliary atresia were seen in female infants, and in males idiopathic neonatal hepatitis was more common (table 1). There were no significant statistical differences between sex and the cause of cholestasis (p=0.069) and also between sex and cause of cholestasis in every diseases diagnosis. 

Onset of jaundice in the study population ranged from 1 to 52 days, and the average age of onset of jaundice was 15.6±16.1 days. The mean age of jaundice onset in biliary atresia compared to idiopathic neonatal hepatitis was lower but not statistically significant (p=0.130). According to the age of the onset of cholestasis in different causes, infants with progressive familial intrahepatic cholestasis, and biliary atresia developed earlier onset of jaundice than in cases with hemochromatosis, cystic fibrosis and galactosemia that presented with later onset of jaundice. There was no significant correlation between the age of jaundice onset and the cause of cholestasis (p=0.463). 

Totally, 54 cases (44.3%) had clay-color stool. Twenty infants (66.7%) out of 30 with diagnosis of biliary atresia and 12 (23.7%) out of 30 patients with idiopathic neonatal hepatitis had clay-color stool, which the difference was statistically significant (p=0.002).Only 9 infants had a family history of liver disease: 3 infants with biliary atresia, one with idiopathic neonatal hepatitis, one with bile duct paucity, one case of hepatic vein occlusion, and 3 infants with unknown diagnosis. 

Fourteen babies had relative parents, 4 of whom had biliary atresia; one case had idiopathic neonatal hepatitis and 5 cases with unknown diagnosis.

**Table 2:** Liver function tests in patients with cholestasis

Diagnosis	Albumin	AST	ALT	Alkaline-.phosphatase	Total bilirubin	Direct bilirubin
Biliary atresia	3.8±0.78	375±355.1	200±129.9	1567±952.6	12.33±3.41	5.8±2.05
Biliary duct paucity	3.7±0.53	281±211.2	146.3±117.74	2013±1563.46	17.07±16.04	6.1±3.4
Idiopathic neonatal hepatitis	4.1±0.62	267±185.6	193.7±142.51	1646.4±953.2	11.7±5.4	6.2±3.2
Progressive familial intrahepatic cholestasis	3.6±0.37	156±130.29	98±98.39	1682.5±367.5	7.9±3.2	3.8±0.89
P. value	0.197	0.128	0.231	0.487	0.218	0.175

The most common physical finding was jaundice that was seen in all patients (100%). Other common physical findings were hepatomegaly that was seen in 92 cases (76.4%) and splenomegaly that was seen in 29 patients (23.8%). Twenty patients (66.7%) with biliary atresia and 23 cases (76.7%) with idiopathic neonatal hepatitis had hepatomegaly (p=0.39); no correlation was found between the presence of hepatomegaly in these two diseases. Just 3 (10%) cases out of 30 patients with biliary atresia and 8 (27%) out of 30 with idiopathic neonatal hepatitis had splenomegaly. There was no correlation between splenomegaly and diagnosis of these two diseases (p=0.095).

The comparison of liver function tests in different causes of cholestasis is shown in table 2. As we seen in table 2 the results of liver function tests are not helpful in determining the causes of cholestasis.

Hepatobiliaryscintigraphy scanning was performed in 21 out of the 122 infants under the study. The results of hepatobiliaryscintigraphy scan in 9 cases in favor of biliary atresia and in 6 of these 9 patients the diagnosis was consistent with the results of liver biopsy. In 9 of 12 infants whose hepatobiliaryscintigraphy scan was excluded the diagnosis of biliary atresia; the results were consistent with liver biopsy. Over all, the results of hepatobiliaryscintigraphy scan were similar to liver biopsy in 71.4% (p=0.356). Hepatobiliaryscintigraphy scan showed 6 cases as real positive and 9 cases as real negative. The overall sensitivity and specificity of hepatobiliaryscintigraphy scan in differentiation between biliary atresia and idiopathic neonatal hepatitis were 66.7% and 75%, respectively.

The diagnosis of biliary atresia in all patients was confirmed with intraoperative cholangiography and all of them underwent Kasai operation.

## Discussion

Jaundice is a common symptom in the first two weeks of life which can be physiologic or due to breast milk. However, if jaundice continues more than two weeks, it indicates a critical emergency situation (11). Cholestasis has been recorded by incidence of 1:2500 live births. Different studies show relatively the same causes for cholestasis in infants and about one third of cholestasis cases were due to extra-hepatic cholestasis that included biliary atresia, choledochal cyst, biliary sludge, and biliary duct stricture (12). Due to the weakness in diagnosis of the cause of hepatitis in infants, the main cause of cholestasis was idiopathic neonatal cholestasis in 1970s but with the advancement of medical science and a diagnostic method it gets less gradually (13).

The cholestasis diagnosis has been a challenge for physicians and the early diagnosis is so important since the effectiveness of treatment is more in early diagnosis. For example, different studies showed that diagnosing biliary atresia in the first 60 days is much better for effectiveness of surgery than in 90 days (-). Even if a specific treatment is not available, an early diagnosis can lead to promptly supportive treatment to reduce the complications of cholestasis like bleeding due to vitamin K deficiency (11).

According to recent studies delay in referral of infants with cholestasis is a fundamental problem in 50% of patients. Lack of knowledge about neonatal cholestasis in primary care centers can cause lot of problems for infants, and physical and mental development of infants can be affected, imposing a high cost on the government, which can be controlled by accurate management and early diagnosis    (17, 18). 

Moreover, family has an important role because in most societies, infants are examined in the first two weeks and then they are referred to hospitals for vaccines and strengthening the immune system in 6-8 weeks. In this time period, parents have a critical role in care giving; therefore, their lack of knowledge can be dangerous for infants (11).

Several studies on evaluation of the causes of cholestasis showed idiopathic neonatal hepatitis and biliary atresia as the most important and common causes of cholestasis (19-21, 24, 25). In our study biliary atresia (24.6%), idiopathic neonatal hepatitis (24.6%), and paucity of intrahepatic bile ducts (13.1%) were the most common causes of cholestasis same as most other studies. 

In a study on 50 infants with cholestasis, idiopathic neonatal hepatitis was diagnosed as the most common cause of cholestasis and biliary tract atresia as the second common cause of cholestasis and cholestasis caused by viral infections (rubella and CMV) was the third cause (26). The results of this research are in line with ours but the lower incidence of infections in our country can be due to meticulous care and maternal health before pregnancy. 

Similarly in another prospective study by Chiang Mai, the first most common cause was biliary atresia (48%) and second one was neonatal hepatitis (33%) like our study (25). In both of these studies, biliary atresia was more common in girls and idiopathic neonatal hepatitis in boys. Also, the clay-color stool was more frequent in patients with biliary atresia than in cases with idiopathic neonatal hepatitis, and hepatobiliaryscintigraphy scan sensitivity was high for differentiating between biliary atresia and idiopathic neonatal hepatitis. 

In Alexandrian and Egyptian studies, 29 cases (41.4%) of 70 infants had idiopathic neonatal hepatitis and 17 cases (24.3%) had biliary atresia as the two most common causes respectively which were similar to our study (24). Although on that study, the onset of jaundice and splenomegaly was helpful in diagnosing the causes of the disease. In our study there was no correlation between the causes of cholestasis and the onset of jaundice (p=0.130), presence of hepatomegaly (p=0.39) and splenomegaly (p=0.095). However, the history of clay-color stool is helpful for differentiate cholestasis causes (p=0.002).

A retrospective study on 205 infants under 6 months of age in Sydney showed that idiopathic neonatal hepatitis with 56 cases (25%) as the most common cause and genetic and metabolic diseases with 46 cases (23%) as the second common cause. Other less common causes were bile duct obstruction (20%), cholestasis related to parenteral nutrition (20%), infections (9%), and paucity of intrahepatic bile ducts (3%) (27). In the present study genetic and metabolic diseases were seen in 8.2%, progressive familial intrahepatic cholestasis in 4.1%, and CMV inflection in 1.6%, respectively. Due to the lack of facilities for advanced metabolic and genetic testing in our center, many metabolic enzyme defects are considered as unknown cause.

To evaluate different methods of distinguishing between idiopathic neonatal hepatitis and biliary tract atresia, a prospective study was performed on 65 infants with cholestasis and final diagnosis of idiopathic neonatal hepatitis and biliary tract atresia in Nemazee Hospital, Shiraz in 2006. Thirty-four cases were girls and 31 were boys; the age of 46 infants with idiopathic neonatal hepatitis was 17-61 days and 19 cases of biliary tract atresia were 18-64 days. The mean age of onset of jaundice was considerably lower in infants with bile duct atresia (p=0.032). The accuracy of different methods were: liver biopsy 96.9%, clinical assessment 70.8%, ultrasound 69.2%, hepatobiliary scintigraphy scan 58.5% and liver enzymes 50.8% (9). In this study, the liver biopsy and clinical assessment were the most reliable methods of differentiation of biliary atresia and idiopathic neonatal hepatitis. In the present study, 30 infants (24.6%) had biliary atresia (onset age of jaundice 1-42 days) and 30 cases had idiopathic neonatal hepatitis (onset of jaundice 1-42 days) and these two diseases were similar in outbreak. Perhaps due to lack of any other center to diagnose cholestasis in the South in 2006 but now having diagnosis and treatment facilities there are likely lower cases referred to this center. Median age of onset of biliary atresia was 3 while it was 10 for idiopathic neonatal hepatitis but there was no significant correlation between them (p=0.130). This can be due to age distribution of patients in our study. Hepatobiliary scintigraphy scan was done for 21 of 122 infants, which was similar to the biopsy results in 71.4% (p=0.356). In our study also there was no significant correlation between the cause of cholestasis and type of liver enzyme elevation. 

The result of our study showed that biliary atresia and idiopathic neonatal hepatitis are the most important causes of cholestasis in this region. Therefore, more attention to them can help physicians to manage patients better and more effectively. In general, the results of this study are consistent with those of previous studies and it seems that the most important factor in managing patients is defining and implementing a regular system for faster diagnosis.

Finally, the limitations of this study are its retrospective nature and the lack of patients’ survival reviews. Due to the sensitive nature of the disease and the need for education of general physicians and pediatricians about the causes of cholestasis and its diagnosis, there is a need for further studies. It is suggested that more attention should be paid to genetic and metabolic disorders and also the facilities should be provided for diagnosis of these diseases in referral centers such as our center.
